# NMDA Receptor C-Terminal Domain Signalling in Development, Maturity, and Disease

**DOI:** 10.3390/ijms231911392

**Published:** 2022-09-27

**Authors:** Kirsty Haddow, Peter C. Kind, Giles E. Hardingham

**Affiliations:** 1 UK Dementia Research Institute, Edinburgh Medical School, University of Edinburgh, Chancellor’s Building, Edinburgh EH16 4SB, UK; 2 Simons Initiative for the Developing Brain, University of Edinburgh, Hugh Robson Building, George Square, Edinburgh EH8 9XD, UK; 3 Centre for Discovery Brain Sciences, University of Edinburgh, Hugh Robson Building, George Square, Edinburgh EH8 9XD, UK

**Keywords:** NMDA receptor, carboxyl (C)-terminal domain (CTD), neurodevelopment, excitotoxicity, neurodegeneration

## Abstract

The NMDA receptor is a Ca^2+^-permeant glutamate receptor which plays key roles in health and disease. Canonical NMDARs contain two GluN2 subunits, of which 2A and 2B are predominant in the forebrain. Moreover, the relative contribution of 2A vs. 2B is controlled both developmentally and in an activity-dependent manner. The GluN2 subtype influences the biophysical properties of the receptor through difference in their N-terminal extracellular domain and transmembrane regions, but they also have large cytoplasmic Carboxyl (C)-terminal domains (CTDs) which have diverged substantially during evolution. While the CTD identity does not influence NMDAR subunit specific channel properties, it determines the nature of CTD-associated signalling molecules and has been implicated in mediating the control of subunit composition (2A vs. 2B) at the synapse. Historically, much of the research into the differential function of GluN2 CTDs has been conducted in vitro by over-expressing mutant subunits, but more recently, the generation of knock-in (KI) mouse models have allowed CTD function to be probed in vivo and in ex vivo systems without heterologous expression of GluN2 mutants. In some instances, findings involving KI mice have been in disagreement with models that were proposed based on earlier approaches. This review will examine the current research with the aim of addressing these controversies and how methodology may contribute to differences between studies. We will also discuss the outstanding questions regarding the role of GluN2 CTD sequences in regulating NMDAR subunit composition, as well as their relevance to neurodegenerative disease and neurodevelopmental disorders.

## 1. Introduction

N-methyl-d-aspartate receptors (NMDAR) are cation-passing channels gated by the principal excitatory neurotransmitter glutamate which play a crucial role within the central nervous system (CNS) [1,2]. They are permeable to the flow of Ca^2+^, K^+^ and Na^+^, with Ca^2+^ being integral to mediating many of the consequences of NMDAR activity, including the intracellular signalling cascades that govern synaptic modification, activity-dependent development, and neuroprotective signalling. Additionally, inappropriate activation of NMDARs forms an important role in part of the pathological processes underpinning excitotoxic cell death and synaptotoxicity in both acute and chronic neurological disorders [3,4,5]. NMDARs are heterotetramers consisting of two obligate GluN1 subunits and two GluN2 subunits (GluN2A-D). Expression of GluN2 subunits is regionally localised, with GluN2A and GluN2B being predominant in the forebrain, GluN2C expressed in the cerebellum and GluN2D in the midbrain [1,2]. The identity of the GluN2 subunits contributes to many of the biophysical properties of the receptor, including agonist affinity, channel open probability, and deactivation kinetics [2,6]. The GluN2 subunits have distinct temporal patterns of expression, compared to GluN2A, GluN2B expression begins earlier in the embryonic brain and maintains high levels of expression during early postnatal development before becoming mainly restricted to the forebrain [7]. After the first two postnatal weeks GluN2A steadily increases, becoming abundant throughout the entire adult CNS. This developmental shift in subunit expression allows for greater GluN2A representation and creates a population of both diheteromeric GluN1_2_-GluN2A_2_ and GluN1_2_-GluN2B_2_ NMDARs as well as triheteromeric GluN1_2_-GluN2A-GluN2B NMDARs [1,2].

The consequences of NMDAR activation do not rely solely on the influx of ions, but also involve interactions between the NMDAR and several signalling molecules and complexes [8]. The binding sites for these signalling/scaffolding proteins are found within the ~600 amino acid sequence that makes up the GluN2A and GluN2B CTD (CTD^2A^ and CTD^2B^) [9]. While GluN2 subunits are well conserved in the N-terminal and transmembrane regions, their CTDs have diverged much more, allowing CTD identity to differentially influence the recruitment of signalling complexes and downstream signalling, trafficking, and the functional diversity of NMDAR signalling [10]. However, there are still outstanding questions as to how CTD identity influences key processes such as activity-dependent changes to NMDAR composition, outcome to excitotoxic insult and its role in chronic neurodegenerative states, as well as its contribution to neurodevelopmental disorders. We will attempt to summarise these questions, both where fresh studies are required and also in areas where there are apparent disagreements in the field.

## 2. The Role of the CTD in Developmental and Activity-Dependent Changes in NMDAR Subunit Composition

The subunit composition of NMDARs changes over the course of development and in response to changes in sensory experience [11,12,13,14,15]. The visual cortex (VC) of rodents has proven to be a particular area of focus in observing activity-dependent changes to subunit composition owing to the ease at which sensory input to the VC can be manipulated. From studies in rats, it has been demonstrated that dark rearing either from birth or during the critical period reduces the levels of GluN2A at the synapse, resulting in a reduction in the ratio of GluN2A to GluN2B (2A:2B ratio) [14,15]. Interestingly, dark reared rats exhibit a rapid increase in GluN2A following 2 h re-exposure to light, suggesting that NMDAR subunit composition, and as such the 2A:2B ratio, can be bidirectionally modified by activity [14,15]. Therefore, the control of this ratio requires a cellular mechanism that can distinguish between GluN2A and GluN2B and selectively incorporate/remove subunits as required within either a developmental or activity-dependent context.

One model for exchanging GluN2B for GluN2A proposes a series of phosphorylation events at a site on the CTD^2B^ [16,17]. NMDAR activity mediates activation of CaMKIIα leading to the formation of a trimolecular complex consisting of GluN2B/CaMKIIα/Casein kinase 2 (CK2). This association of CK2 with GluN2B leads to phosphorylation of serine-1480 (S1480) on CTD^2B^. Phosphorylation of S1480 leads to disrupted association of membrane-associated guanylate kinase (MAGUK) proteins at the CTD^2B^ PDZ ligand and a subsequent reduction in phosphorylation at Y1472 within the YEKL endocytic motif, due to the fact that MAGUKs ordinarily recruit the Y1472 kinase Fyn. These events are suggested to lead to lateral diffusion of GluN2B to extrasynaptic sites via a non-PDZ interaction with SAP102 before they are internalised by AP-2 mediated endocytosis, which recognises YEKL when Y1472 is unphosphorylated [16,17,18]. However, this model was based on experiments involving ectopic overexpression of mutant subunits, potentially altering the relative stoichiometry of CTD^2B^ and CaMKIIα or other signalling components that may affect the results. Subsequently, a KI mouse model with a non-functional CaMKIIα binding site (GluN2B^∆CaMKII^) was generated that had normal synaptic levels of GluN2A in the adult hippocampus suggested that mutating the CaMKIIα binding site may at most delay the developmental subunit shift [19]. Moreover, an independently made second KI mouse with a non-functional CaMKIIα binding site (GluN2B^∆CaMKII^) showed that the CaMKIIα site was not required for normal developmental increases in 2A:2B ratio [20]. However, this study only tested the role of CaMKIIα site in developmental subunit changes and as such did not rule out the possibility that this site may be important in activity-dependent subunit changes. The same study also utilised a KI mouse model with the CTD of GluN2A replaced by that of GluN2B (GluN2A^2B(CTR)^) [10] and found that changes to the NMDAR composition in the cortex and hippocampus during development was normal and so do not require GluN2A and GluN2B to have distinct CTDs [20].

Interestingly, while the above study shows that the CTD of GluN2A can be swapped for that of GluN2B with no impairment of GluN2A surface expression, recent studies suggested that in the context of the CTD^2A^, certain residues may be functionally important for surface expression of GluN2A. Work by Mota Vieira et al. [21] and Yong et al. [22] aimed to functionally characterise the epilepsy- associated variant GluN2A-S1459G and identified S1459 as a CaMKIIα phosphorylation site controlled in a development and activity-dependent manner whose mutation impaired interactions with SNX27 and PSD95 as well as GluN2A surface expression when ectopically expressed in cultured neurons. Generation and characterisation of a GluN2A-S1459G KI mouse would further strengthen the case for this phosphorylation event being functionally important for activity-dependent and/or developmental surface expression of GluN2A.

Overall, there are two theories here by which subunit composition shifts during development. The studies which propose the involvement of key CTD phosphorylation events are based predominantly in vitro using heterologous expression of mutant receptors, and as such would benefit from in vivo studies involving KI mice. The in vivo models used thus far have argued against a critical role for the CaMKIIα site of CTD^2B^ and subsequent phosphorylation changes proposed by in vitro models. If it is the case that phosphorylation events are not crucial, what are the molecular mechanisms that underpin this change? It is an under-appreciated fact that even in the adult mouse the forebrain contains far more GluN2B than GluN2A [23]. The dramatic increase in GluN2A during development, alongside flat or falling levels of GluN2B can at first glance appear like a subunit “switch”—a term that is often used. However, by exploiting KI mice with reciprocal exchange of GluN2 CTDs it was demonstrated that GluN2B levels are around 5-times greater than GluN2A [23]. This means that increased GluN2A insertion is likely to be the main driver of 2A:2B ratio changes since removal of GluN2B on its own would have little effect on the ratio unless very large reductions in GluN2B were involved. Indeed, the 2A:2B ratio has been shown to change developmentally with little or no change to GluN2B levels [20]. This argues against CTD^2B^-dependent removal of GluN2B being biologically important. The reason why this modest level of GluN2A (even in the adult) has such a strong effect on NMDAR currents (as evidenced by sensitivity to GluN2A antagonists or reduced sensitivity to GluN2B antagonists [24,25]) is that GluN1_2_-GluN2A_2_ diheteromeric NMDARs have a higher open probability than GluN1_2_-GluN2B_2_ diheteromeric NMDARs and, moreover, GluN1_2_-GluN2A-GluN2B triheteromeric NMDARs more closely resemble GluN1_2_-GluN2A_2_ NMDARS than GluN1_2_-GluN2B_2_ NMDARs with regard to their biophysical properties [26]. If removal of GluN2B plays a limited role in the developmental change of 2A:2B ratio and insertion of GluN2A is more important, then what of the role of CTD^2A^? Since the developmental 2A:2B ratio shift occurs normally in GluN2A^2B(CTR)^ mice where both GluN2B and GluN2A have the same CTD (CTD^2B^) [20] then CTD^2A^-dependent events cannot be critical for GluN2A surface delivery. Since GluN2A expression at the mRNA and protein level increases at the same developmental stage as the 2A:2B ratio increases, it could be that this is the only change required, coupled with normal turnover of synaptic proteins.

Experience/activity dependent changes in 2A:2B ratio represent a different situation, studied in most depth in the VC. If sensory deprivation (e.g., dark rearing) causes the 2A:2B ratio to fall, and rise again upon re-exposure to light, then these dynamic changes arguably do require a mechanism for recognising GluN2A-containing NMDARs in instances where they are lost/gained on a timescale incompatible with receptor turnover and changes in GluN2A gene expression (GluN2A transcription can be controlled by synaptic activity to influence slower-acting changes [27]). Investigations into such ‘recognition’ mechanisms will require further analysis of current KI mouse models as well as new ones, potentially with more targeted mutations in CTD^2A^ or other cytoplasm-facing regions (i.e., the loops that link the membrane spanning domains).

## 3. GluN2A/2B CTD Mutations Associated with Neurodevelopmental Disorders

**Figure 1:**
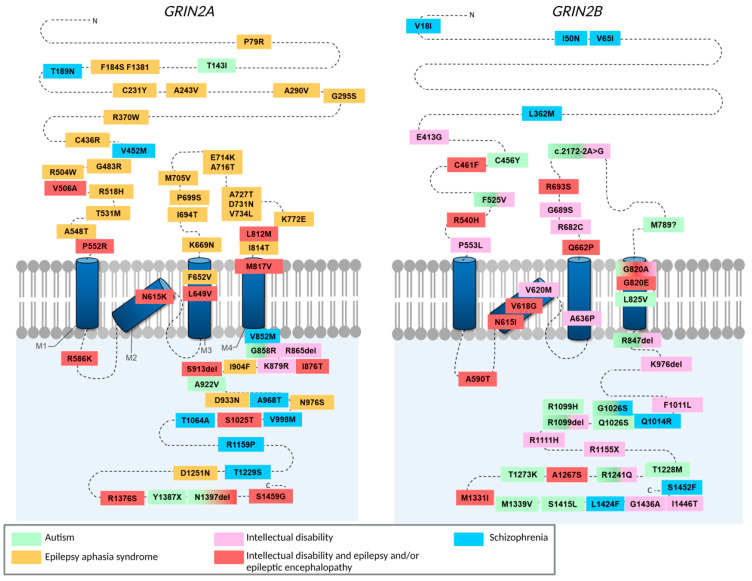
Schematic showing the locations of heterozygous missense, nonsense and frameshift mutations in *GRIN2A* (glutamate receptor ionotropic NMDA 2A) and in *GRIN2B* (NMDA 2B) that have been identified in people with neurodevelopmental disorders. The extreme extracellular amino terminus of these subunits contains allosteric modulation sites. The region between the N terminus and the M1 domain, plus the extracellular loop between the M3 and M4 domains, encode the glutamate-binding domain. The M2 domain features many side chains that point towards the receptor channel pore and dictate ion permeability. Finally, the long cytoplasmic carboxy-terminal domain is involved in receptor targeting and coupling to downstream signalling complexes. Figure based on refs. [21,29,30,31,32,33,34,35,36,37,38,39,40,41,42,43,44,45,46,47,48,49,50,51,52].

Genome sequencing studies have identified over 200 neuropathology associated variants in GRIN genes, with a large proportion of these variants being found in *GRIN2A* and *GRIN2B* (Figure 1). As may be expected, most of these variants are found within the highly conserved amino binding domain (ABD) and transmembrane domains (TMD), however, ~20% of these variants occur within the CTD (reviewed by [28]). Mutations are being discovered at a faster rate than the labour-intensive functional characterisation of these CTD variants, but some variants associated with autism spectrum disorder (ASD), intellectual disability (ID) and epilepsy have been studied in detail.

Liu et al. [41] characterised an autism associated GluN2B mutant occurring in the CTD of GluN2B (S1415L human, S1413L rodent). Rat hippocampal neurons transfected with mutant GluN2B showed a 30% reduction in receptor surface expression compared to GluN2B WT. In addition, GluN2B S1413L-expressing neurons also showed a reduction in spine density. From this it would appear that the CTD^2B^ plays an important role in the trafficking of GluN2B to the synaptic membrane which when impaired may lead to a reduction in dendritic spines. For CTD^2B^ mutations in which trafficking deficits and morphological changes are observed, there is some question as to whether differences in morphology occur as a result of the loss of intracellular signalling linked to the CTD^2B^ or whether these changes are simply a consequence of altered NMDAR at the synapse. Using chimeric mouse models with reciprocally swapped CTDs, Keith et al. [53] found that replacing CTD^2A^ for CTD^2B^ in GluN2A results in longer total dendritic path, average apical length, and total basal length relative to WT mice. They observed no difference between WT and a model replacing CTD^2B^ for CTD^2A^ in GluN2B, and importantly, replacing the ABD or TMD of GluN2A for that of GluN2B had no effect on morphology. As such, these results indicate that CTD^2B^-specific intracellular signalling pathways are a key regulator in dendritic morphology, raising the possibility that deficits in CTD^2B^ signalling may contribute to altered development in ASD/ID. Future studies probing the potential role of the S1423L variant on dendritic complexity and spine morphology will help to shed light on the role of CTD^2B^ signalling in ASD/ID neurodevelopment [41].

Mutations in *GRIN2A* are commonly associated with developmental delay and epileptic phenotypes [38,54]. While these mutations predominantly occur in the ABD or TMD resulting in functional changes to the subunits, a number of epilepsy-associated variants have also been identified within the CTD^2A^ [21,38,39]. In addition to the NMDAR mediated Ca^2+^ influx that is required for the induction of plasticity, there is also some evidence to suggest that GluN2 CTD identity influences synaptic plasticity induced by different patterns of activity [10]. Therefore, mutations that either affect trafficking of the GluN2A subunit or that alter key interaction sites have the potential to impair synaptic plasticity. However, seemingly counterintuitively, mutations in the CTD^2A^ that enhance trafficking also negatively impact plasticity. Li et al. [40] recently characterised a rare ID associated variant in the GluN2A subunit. The mutation, K879R, was found to occur within an endoplasmic retention signal motif and resulted in enhanced cell surface expression. The increased expression of GluN2A led to deficits in synaptic transmission both in GluN2A_K879R mouse hippocampal CA1 neurons and in K879R KI mice. Additionally, KI mice exhibited impairments in the induction of LTP and LTD as well as deficits in learning and memory. This suggests the importance of a carefully controlled balance of GluN2A levels, or 2A:2B ratio. It also suggests that any therapeutic approaches should avoid the potentially severe impacts of over-correcting GluN2A deficits.

As mentioned previously different GluN2 subunits influence the biophysical properties of NMDARs. The relatively faster deactivation kinetics of GluN2A-containing NMDARs relative to GluN1_2_-Glun2B_2_ NMDARs allows for GluN2 subunit composition to modify Ca^2+^ influx in response to synaptic input and as such the downstream consequences such as the induction of LTP vs. LTD. Furthermore, it has been observed that altering synaptic activity leads to changes in neural function that influences the subsequent induction of synaptic plasticity—so called “metaplasticity” (reviewed thoroughly elsewhere [55,56]). As such, control of the 2A:2B ratio in response to synaptic input has been proposed as one of the mechanisms by which neuronal activity is kept within a functional range in response to changing levels of activity. Considering the potential role of NMDAR subunit composition in homeostatic regulation of activity, it could be suggested that mutations in *GRIN2A* promote epileptic phenotypes through either a reduction or loss of function of the GluN2A subunit itself, e.g., caused by mutations that affect agonist binding and channel properties, or through a reduction in subunit expression at the synapse. The result of any of these mutations would lead to reduction in GluN2A activity, thereby leading to an impaired homeostatic ability of circuits and the facilitation of hyper-excitability. However, it should also be noted that a loss of excitatory drive from inhibitory interneurons and subsequent disinhibition as a result of a loss of GluN2A function may also contribute to hyper-excitability. Indeed, it was recently shown that enhancing GluN2A activity in both excitatory and inhibitory interneurons with the use of the positive allosteric modulator (PAM) GNE-0723 reduced epileptiform activity in mouse models of Alzheimer’s disease (AD) and Dravet syndrome [57]. Overall, it appears that the 2A:2B ratio is important in mediating seizure susceptibility. Specifically, a decrease in this ratio favours increased seizure susceptibility. In the hippocampus both a decrease in GluN2A expression [58] and upregulation of GluN2B [59] have been implicated in contributing to seizure pathogenesis. In addition, selective blockade of GluN2B has been shown to reduce seizure susceptibility [60,61] and dextromethorphan induced convulsive behaviours [62]. However, it remains unclear whether the beneficial effect of modifying the relative activity of GluN2A/2B is solely the result of restoring homeostatic balance or whether subunit specific signalling pathways are also involved. Crucially, there is currently no evidence to suggest that CTD-dependent effects mediate the subunit-specific effect on seizure pathogenesis.

As advances in the technology make it easier to generate genetic models for *GRIN2A* variants, the challenge shifts to assessing the face validity of these models. For example, a recent study generated a KI mouse for a de novo heterozygous *GRIN2A* variant identified in a patient with ID and epileptic encephalopathy (S644G) [63]. Mice homozygous for S644G unexpectedly died of a lethal seizure during the third postnatal week, but heterozygous mice had a normal lifespan and exhibited neither behavioural seizures nor epileptiform activity. They did, however, exhibit both an increased seizure susceptibility and an increased seizure resistance in response to different electroconvulsive threshold testing, highlighting the biological complexity of *GRIN2A* involvement in epileptic encephalopathy [63]. Therefore, while mouse models provide a valuable opportunity to probe the cellular and circuit level consequences of *GRIN2A* mutations, care should be taken when translating findings across species.

Overall, understanding the molecular consequences of *GRIN2A* and *GRIN2B* mutations may provide an opportunity for targeted therapeutic strategies. Indeed, several preclinical pharmacological studies have highlighted the potential for the use of both negative allosteric modulators (NAMs) and PAMs in treating gain of function and loss of function *GRIN* variants, respectively [64,65,66,67]. However, it is important to consider that allosteric modulators may be influenced by intracellular factors such as the phosphorylation, ubiquitination and palmitoylation states of CTDs. This was highlighted in a recent study in which it was demonstrated that GluN2 deletions robustly altered the activity of both PAMs and NAMs, likewise, agents altering phosphorylation state and intracellular Ca^2+^ levels were also observed to produce receptor-specific and compound specific changes to PAM activity [68]. Therefore, further studies investigating the metabotropic influence of CTDs on the activity of allosteric modulator will be a crucial step in paving the way to developing appropriate treatment strategies for aberrant NMDAR activity.

## 4. A Critical Role for CTD Interactions in Acute Excitotoxicity

**Figure 2:**
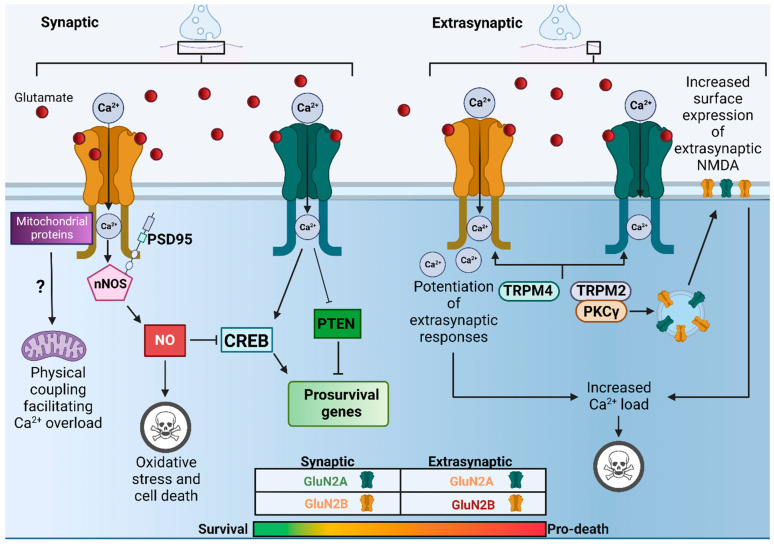
Schematic showing some of the proposed mechanisms underpinning CTD specific and location specific downstream consequences of acute excitotoxicity. At synaptic sites activation of GluN2A containing NMDARs (green) promotes expression of CREB and pro-survival genes and inhibits the PTEN pathway shut-off of pro-survival genes. Synaptic GluN2B containing NMDARs (yellow) may mediate pro-survival or pro-death signalling depending on the level of glutamate. When faced with excitotoxic glutamate levels preferential GluN2B/PSD95/nNOS coupling promotes NO mediated shut off of CREB, oxidative stress and subsequent cell death. Direct coupling of CTD^2B^ to mitochondrial proteins may also facilitate Ca^2+^ overload and mitochondrial dysfunction (potential but unconfirmed pathway; ?). At extrasynaptic sites physical and functional coupling of both CTD^2A^ and CTD^2B^ with TRPM2 and 4 may result in enhanced extrasynaptic NMDAR activity to promote cell death. Therefore, it is possible that a hierarchy exists in which a combination of both composition and location determines the downstream consequences of NMDARs.

It has been observed that when neurons are subject to sustained elevated glutamate, cell death occurs [69]. In the 1980s, it was observed that excessive Ca^2+^ influx through NMDARs is a key mediator in the neuronal death observed in response to glutamate exposure [70]. Moreover, Tymianski et al. [71] demonstrated that the route of entry, specifically via NMDARs, was more important than overall Ca^2+^ load. This finding was later expanded on by Sattler et al. [72] who confirmed that lower Ca^2+^ influxes through NMDARs produced greater lethality when compared to high Ca^2+^ influx via other Ca^2+^ permeant channels. As such, these findings suggest that there is either functional and/or physical coupling of NMDARs to Ca^2+^-responsive mediators of cell death signalling. Of note however, physiological patterns of synaptic NMDAR activity are known to be protective leading to the classic bell-shaped curve model for neuronal response to NMDAR activity [73,74]. However, beyond simple level of activity mattering, the question remained as to whether NMDAR subunit compositional diversity influences the consequence of NMDAR activation, particularly via the divergent CTDs of GluN2A and GluN2B. GluN2A and GluN2B containing NMDARs are both capable of mediating excitotoxicity; however, there is evidence to suggest that CTD identity may influence the tendency to couple to either pro-death or pro-survival signalling pathways (Figure 2). Of note, there is also emerging evidence for the presence of ion flux independent excitotoxicity. Recently, it has been reported that NMDARs form a signalling complex with Src kinase and Panx1 in response to NMDAR ligand binding without activation of their ion conduction pore [75]. Furthermore, disruption of this signalling complex was observed to be neuroprotective both in vitro and in vivo [75]. This is somewhat controversial as it disagrees with many of the existing studies regarding the ion flux dependent nature of excitotoxicity. Regardless of this, as ion flux-independent mechanisms appear to involve NMDAR recruitment of signalling complexes it would be of interest to establish the role of GluN2 CTD specific interactions in this proposed mechanism for excitotoxicity.

Previously, it has been demonstrated that GluN2A containing NMDARs are linked to intracellular signalling cascades that promote the activation of neuroprotective transcription factors such as cAMP response element-binding protein (CREB) and a reduction in the expression of genes such as phosphatase and tensin homolog (PTEN), which is involved in pathological processes associated with neuronal injury [76,77]. In contrast, GluN2B containing NMDARs have been shown to couple with signalling pathways that supress CREB-dependent survival pathways [78]. However, it is important to note that the CTD^2B^ can also mediate signalling that is neuroprotective. For instance, the GluN2B-PTEN signalling pathway has also been shown to enhance the expression of Dysfunction of PTEN-induced kinase 1 (PINK1) which potentiates GluN2A and its pro-survival signalling pathways [79].

In addition to the preferential recruitment of pro-death vs. pro-survival signalling mediated by GluN2 CTD identity, there is also evidence to suggest that location rather than composition alone is a key determinant in whether pro-death signalling pathways are preferentially activated. For instance, it has been demonstrated that extrasynaptic NMDARs preferentially promote CREB inactivation and mitochondrial dysfunction whereas activation of their synaptic counterparts does not induce mitochondrial dysfunction and instead induces protective CREB-dependent gene expression in a nuclear Ca^2+^-dependent manner [3,78,80,81,82,83,84,85]. Interestingly, this distinction between extrasynaptic and synaptic NMDARs persists during developmental time points where GluN2B is predominant, suggesting that location is a significant factor in determining the downstream consequences of NMDAR activation [86,87]. Overall, a combination of both identity and location are likely to be important in determining the downstream consequences of NMDAR activation, although it will be of benefit in the future to validate key findings in human neuronal models of excitotoxicity including strategies to alter synaptic-extrasynaptic balance [88,89].

### 4.1. GluN2B Mediated Excitotoxicity through PSD95-nNOS Pathway

Using the novel GluN2A^2B(CTR)^ and GluN2B^2A(CTR)^ KI models Martel et al. [90] observed that GluN2A^2B(CTR)^ mice showed enhanced NMDA induced excitotoxicity compared to WT. Interestingly, it was also found that GluN2B^2A(CTR)^ mice showed a reduction in the vulnerability of forebrain neurones to excitotoxic levels of Ca^2+^ influx through the NMDAR both in vivo and in vitro. Thereby suggesting that GluN2B may preferentially couple to pro-death signalling pathways. Indeed, when examining the mechanistic basis for GluN2 CTD subtype specific differences, it was observed that GluN2B^2A(CTR)^ mice exhibited both a prolonged phosphorylation of CREB and a reduction in coupling to nitric oxide (NO) production in response to excitotoxic conditions. NO can supress CREB phosphorylation at high levels [91,92,93], it is produced when NMDAR-mediated Ca^2+^ influx activates nitric oxide synthase, which is recruited to the NMDAR signalling complex via PSD95 association with CTD^2B^ [91]. Therefore, the findings by Martel et al. [90] suggest that CTD^2B^ may preferentially couple to this PSD95/nNOS pathway to promote cell death.

In light of the failure of conventional NMDAR antagonists in stroke trials [94], there is a need for more finely tuned interventions that can target specific pro-death signalling interactions without impairing physiological functioning of NMDARs. The generation of a cell-permeable mimetic peptide of the GluN2B-PSD95 PDZ domain, known as NA-1 (also known as TAT-NR2B_9C_), that was designed to reduce coupling of nNOS to GluN2B via PSD95 produced a lot of optimism owing to its neuroprotective effects in stroke models in rodents and monkeys [91,95]. However, following on from a successful phase II trial for safety and efficacy for iatrogenic micro-strokes during cerebral aneurysm repair, it has since failed human clinical [96,97]. However, an exploratory analysis did reveal a potential drug–drug interaction between NA-1 and the usual care thrombolytic alteplase that may nullify the treatment effect of the former [96]. This observation requires further investigation, but it leaves some hope for the therapeutic benefit of NA-1.

### 4.2. The Role of Extrasynaptic Specific Physical and Functional Coupling in Excitotoxicity

It has been suggested that extrasynaptic specific protein(s) coupling to the NMDAR promotes pro-death signalling pathways. Yan et al. [98] identified a physical interaction between extrasynaptic NMDARs and TRPM4 that they demonstrated as being vital in facilitating excitotoxicity. The use of small molecule NMDAR/TRPM4 interaction interface inhibitors was observed to provide robust protection against cell death in vivo and in vitro as well as inhibiting excitotoxicity-mediated transcriptional changes and mitochondrial dysfunction. Since proteomic data from mouse and human cortex and hippocampus indicates that TRPM4 is absent at the synapse, this study proposes that NMDAR-TRPM4 interactions may offer some explanation as to the differences in the response of synaptic vs. extrasynaptic NMDARs during increased Ca^2+^ load. Similarly, it has also been observed that TRPM2 ion channels functionally couple with extrasynaptic NMDARs to enhance excitotoxicity in mouse models of ischaemic brain injury [99]. *Trpm2* knock-out (KO) mice exhibited a reduced infarct volume and an improved neurological performance compared to WT mice following middle cerebral artery occlusion (MCAO), which is in agreement with observations made previously in these KOs [100]. Furthermore, it was found that Protein kinase C gamma (PKCγ) was readily coimmunoprecipitated with anti-TRPM2 in brain lysates and that this interaction was increased following MCAO [99]. Considering that PKCγ has been shown to regulate NMDAR surface trafficking [101,102], this study suggests that TRPM2-NMDAR interactions may exacerbate excitotoxicity by increasing the surface expression of extrasynaptic NMDARs, enhancing extrasynaptic NMDAR activity thereby promoting cell death.

Both studies observe a reduction in cell death following uncoupling of their respective TRPM channels, suggesting that uncoupling of either would to be sufficient to reduce cell death. This implies that both TRPM2 and 4 are required for extrasynaptic mediated cell death, potentially with one interaction mediating increased levels of extrasynaptic NMDARs and the other mediating location specific potentiation of extrasynaptic NMDAR responses. Interestingly, both studies demonstrated that TRPM2 and TRPM4 interaction sites are present on both CTD^2A^ and CTD^2B^. This coupled with the observation by Martel et al. [90] that swapping the CTD^2B^ with that of CTD^2A^ reduces but does not abolish excitotoxicity, further suggests that CTD identity may be an additive factor to the influence of location (synaptic vs. extrasynaptic) on excitotoxicity.

### 4.3. DAPK1 Interactions at CTD^2B^ Do Not Mediate Excitotoxic Cell Death

While earlier work by Tu et al. [103] suggested a role for death-associated protein kinase 1 (DAPK1) mediated phosphorylation of CTD^2B^ Ser-1303 (s1303) in the pro-death signalling that occurs during excitotoxicity, subsequent studies have failed to reproduce these findings. Indeed, it has since been observed that excitotoxicity persists even in the absence of DAPK1, in vivo and in vitro, and moreover, s1303 phosphorylation does not differ between neuronal cultures from DAPK1^−/−^ and DAPK1^+/+^ mice during either basal or excitotoxic conditions [104]. In agreement with this, another study using GST-fusion proteins with the CTD of GluN2B as a phosphorylation substrate for CaMKIIα and DAPK1 found that phosphorylation of s1303 is four orders of magnitude weaker by DAPK1 compared to CaMKIIα and therefore s1303 is a poor substrate for DAPK1 phosphorylation [105] (Table 1). Based on these finding, CTD^2B^-CaMKIIα interaction would appear to be a better candidate mechanism in CTD^2B^ mediated excitotoxic cell death. If this were to be the case, one would predict that mice such as the GluN2B^∆CaMKII^ mouse, which possess a mutated CaMKIIα binding site, would exhibit a reduction in cell death when compared to their WT counterparts. In agreement with this, Buonarati et al. [106] demonstrated that ΔCaMKII mice (L1298A/R1300Q) exhibit reduced hippocampal cell death following an in vivo cardiac arrest/cardiopulmonary resuscitation model (CA/CPR). Crucially, while the ΔCaMKII mutation abolished CaMKIIα binding at CTD^2B^, no affect was observed for DAPK1. Therefore, based on the current evidence, it would appear that CTD^2B^-DAPK1 binding is not a key mediator of excitotoxic cell death.

When looking for an explanation for contradictory findings between the initial study and subsequent studies, attention falls to the methodology. Tu et al. [103] demonstrated a neuroprotective effect by using a peptide mimetic of S1303, Tat-NR2B_CT_, to uncouple CTD^2B^-DAPK1 interaction. However, McQueen et al. [104] found that the Tat-NR2B_CT_ acts as a potent NMDAR antagonist and hypothesised that this was due to the peptide binding near the Mg^2+^ site. In agreement with this, they found that Tat-NR2B_CT_ acted as an open channel blocker. Therefore, this may account for the neuroprotective qualities previously observed. In addition, the study by Tu et al. [103] also used a 2,3,5-Triphenyl tetrazolium chloride (TTC) stain as a measure for brain infarct volume. TTC is a water-soluble dye that is reduced by mitochondrial enzymes, as such it reflects metabolic consequences of ischaemia that may occur independently of cell death [107,108]. Therefore, the reduced infarct volumes observed in response to DAPK1 deletion may reflect reduced metabolic damage rather than cell death. In agreement with this, it was concluded by a recent meta-analysis of the literature that while inhibition of DAPK1 does reduce the gradual loss to neuronal function and structural integrity (i.e., degeneration) following cerebral hypoxic ischaemia, it does not alter the immediate cell death observed in response to ischaemia [109]. Therefore, while DAPK1 may play a role in the mechanisms that lead to the impairment of neuronal function following ischaemia, other pathways mediate ischaemic cell death.

### 4.4. Role of GluN2A and GluN2B in Tissue-Type Plasminogen Activator Mediated Neuroprotection

Recent evidence also suggests a role for subunit identity in differentially mediating the downstream consequences of non-conventional NMDAR binding proteins. Tissue-type plasminogen activator (tPA) is a serine protease involved in the breakdown of blood clots, as such, it is used in the early stage treatment of ischaemic stroke in order to aid the breakdown of clots and restore blood flow. Previously it has been demonstrated that tPA interacts with the ABD of GluN1 to promote a selective increase in the surface dynamics of extrasynaptic NMDARs resulting in the promotion of NMDAR-mediated calcium influx and excitotoxicity [110]. However, tPA interaction with GluN2A containing NMDAR has been shown to promote neuroprotective signalling, an effect which is ameliorated by blocking either GluN2A or synaptic NMDARs [111,112]. Bases on the previous assumption that GluN2B is enriched at extrasynaptic sites, this finding seemed to indicate differential roles for tPA interaction with GluN2A and GluN2B in mediating neuroprotection and pro-death signalling, respectively. However, current evidence suggests that GluN2 composition may not be drastically different between synaptic and extrasynaptic sites [90,113,114,115], again suggesting that the main driving force in the loss of neuroprotection is the activation of extrasynaptic-specific pathways, with subunit composition possibly providing an additive effect. Interestingly, a recent study demonstrated that the two-chain form of tPA (tc-tPA) recruits GluN2B containing NMDARs in a MET receptor tyrosine kinase dependent manner to drive the down regulation of extrasynaptic GluN2B and promote neuroprotection [116]. It is therefore possible that the downstream consequences of tPA interaction with NMDARs depend on a combination of factors including NMDAR location, composition, and the form of tPA involved (single vs. two chain form). Of note, tc-tPA was also observed to reduce CTD^2B^ phosphorylation [116], as such it would be of interest to probe the role of the CTD in tAP mediated neuroprotection using both ΔCaMKII and CTD KI mouse models.

## 5. Role of CTD^2B^ in Alzheimer’s Disease?

The role of CTD signalling in chronic neurodegenerative disease is an area of particular interest, especially with regard to AD. In the AD brain, as the disease progresses, mitochondrial dysfunction and subsequent bioenergetic failure, glutamate release from inflammatory cells and astrocytes, and a reduction in astrocytic glutamate transporter expression may all facilitate the loss of glutamate homeostasis, leading to a rise in ambient glutamate [117,118,119]. As opposed to the rapid cell death that is observed during acute excitotoxicity, this chronic increase in ambient glutamate may act as a low-level, gradual excitotoxic insult that progressively impairs synaptic integrity and eventually leads to cell death [118]. Furthermore, the tonic activation of extrasynaptic NMDARs exacerbates the situation by promoting amyloid-precursor protein (APP) processing [120,121]. Given that there is evidence to suggest that CTD identity influences coupling to pro-death vs. pro-survival signalling in acute excitotoxicity, it is important to question whether CTD identity similarly influences signalling pathways in neurodegeneration.

### 5.1. Role of CTD^2B^ in Ionotropic and Metabotropic Dependent Pathways in AD

The downstream consequences of NMDAR activation have been robustly demonstrated to rely on Ca^2+^ influx via NMDARs, either through increasing intracellular Ca^2+^ or by activation of Ca^2+^ dependent enzymes associated with NMDAR CTDs. However, in recent years there has been some evidence to suggest that at least some consequences of NMDAR activation may be mediated by ion-flux independent metabotropic pathways (reviewed by [122]). So far, it has been demonstrated that ion-independent conformational changes of the CTD alter protein binding in response to NMDA activation in both synaptic plasticity and Aβ pathology [123,124,125,126]. In the context of AD, CTD^2B^ has been implicated as being important in both enhanced ionotropic-dependent pro-death pathways as well as Aβ dependent metabotropic pathways [124].

**Figure 3:**
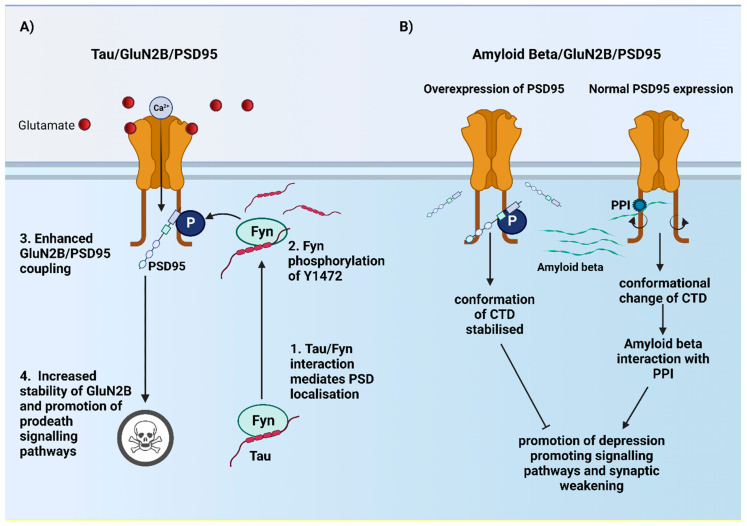
Schematic showing the contrasting mechanisms proposed for the CTD^2B^ in tau and Aβ pathology. (**A**) Interaction between tau and Fyn mediates Fyn localisation to the PSD where it phosphorylates Y1472. Phosphorylation of Y1472 leads to stronger GluN2B/PSD95 coupling resulting in increased GluN2B containing NMDAR stability at the PSD and recruitment of pro-death signalling pathways. (**B**) Enhanced GluN2B/PSD95 coupling either by overexpression of PSD95 or enhancing endogenous expression stabilises the conformation of the CTD^2B^, preventing Aβ interactions with PPI that result in depression and subsequent weakening of synapses.

Using a mouse model expressing a truncated form of tau, ∆Tau^74^, Ittner et al. [127] demonstrated that tau interaction with the SRC kinase Fyn is crucial in determining its localisation at the PSD. Furthermore, they found that localisation of Fyn at the PSD via its interactions with tau led to phosphorylation of the Y1472 site on the CTD^2B^ resulting in a strengthening of the interactions between CTD^2B^ and PSD95. As discussed previously, the interaction between CTD^2B^ and PSD95 is thought to play a crucial role in pro-death signalling, and as such Ittner et al. [127] suggest that tau-Fyn phosphorylation of CTD^2B^ contributes to tau pathology by increasing stability of the NMDARs at the PSD and coupling to excitotoxic downstream signalling pathways (Figure 3A).

In contrast to the findings of Ittner et al. [127], it has also been demonstrated that CTD^2B^-PSD95 interaction may be protective in Amyloid beta (Aβ) pathology. By using organotypic hippocampal slices infected with viral vector containing CT100 (beta-secretase cleavage product of APP) and PSD95 it has been observed that increased interaction of CTD^2B^ and PSD95 may stabilise NMDARs resulting in NMDAR induced potentiation that acts to compensate for Aβ induced depression. GluA1 KO mice fail to exhibit potentiation in PSD95 overexpression slices, thereby demonstrating that PSD95 mediated potentiation requires GluA1. On the other hand, expressing CT100 was still able to mediate depression, however, this depression was abolished by coexpressing PSD95. Taken together, this suggests that PSD95 overexpression blocks Aβ induced depression independently of its ability to potentiate synaptic transmission [124]. From this it was hypothesised that the protective effect of PSD95 may be achieved by constraining the conformation of the CTD such that signalling pathways that promote depression are blocked. Using FRET-FLIM assay, they demonstrated that over expression of PSD95 prevents the reduced FRET efficiency observed in the presence of CT100, and interestingly, it was also observed that overexpression of PSD95 blocked the loss of FRET interaction between GluN1 and protein phosphatase 1 (PP1) [124]. Therefore, they propose a mechanism by which over-expression or increased endogenous expression of PSD95 stabilises CTD^2B^ conformation to prevent Aβ interaction with PPI that promotes synaptic weakening (Figure 3B).

These two studies present two different potential roles for CTD^2B^-PSD95 interactions in the pathology of AD, opposing in nature. The evidence of both a beneficial and detrimental role for CTD^2B^-PSD95 in AD raises many questions, for instance, is one mechanism dominant over the other? That is to say, would the beneficial effect of uncoupling CTD^2B^-PSD95 on tau pathology produce an overall therapeutic effect despite possibly exacerbating Aβ pathology? Another question is whether there are specific time points of the disease pathology in which targeting CTD^2B^-PSD95 interactions would be most beneficial? For instance, while enhancing CTD^2B^-PSD95 interactions might provide some therapeutic effect in early stages of the disease, it may prove detrimental when there is a loss of glutamate homeostasis, potentially contributing to local synaptotoxicity or neuronal loss.

### 5.2. Implications for Astrocytic NMDA Subunits in AD?

As alluded to previously, AD possesses a complex pathology that involves dysfunction of both neurons and glia. Moreover, as NMDARs have also been shown to be expressed in astrocytes [128,129] this yields the possibility that NMDAR activation may contribute to pathological changes observed to astrocytic function. For instance, GluN2C is expressed by astrocytes in the majority of the telencephalon [130,131] and GluN2A has been observed to be expressed in hippocampal astrocytes [132]. Moreover, it was recently demonstrated that astrocytic GluN2A expression is increased in post-mortem brain tissue taken from AD patients [132]. While the exact role of astrocytic NMDARs in disease pathology remains unclear, there is some evidence to suggest that these receptors can mediate both protective and pathological processes. Previous in vitro studies have found that exposure of neuron astrocyte co-cultures to NMDA promotes an increase in the antioxidant capacity of neurons [133] whereas overexposure to NMDA disrupts astrocytic homeostatic function [134,135]. This yields the interesting possibility that the level of astrocytic NMDAR expression and/or subunit composition and CTD identity dependent signalling may contribute to the pathological processes observed in AD and other neurodegenerative disorders in which glial dysfunction is a component. As such, further research is needed to uncover the mechanisms by which astrocytic NMDAR activation may contribute to disease pathology.

## 6. Contribution of CTD^2A^ and CTD^2B^ in Other Disease Pathologies?

As well as AD, there is also evidence to suggest that GluN2 CTD identity contributes to pathology in a wide range of diseases. In the case of Parkinson’s disease (PD), it has been observed that rat models mimicking both early and advanced stage PD exhibit increased synaptic GluN2A, resulting in an increased 2A:2B ratio and plasticity deficits [136,137]. Interestingly, the use of permeable protein mimetics to modulate CTD^2A^ MAGUK binding was found to restore synaptic plasticity and reduce motor impairments [136]. This suggests that CTD^2A^ may serve as a potential therapeutic target in PD, however, as dopaminergic denervation appears to be important in driving subunit compositional changes it would also be of interest to establish the mechanisms by which this occurs. 

GluN2 subunit composition has also been implicated in Huntington’s disease (HD). Of note, it has been observed across multiple species that the striatum exhibits higher levels of GluN2B relative to other brain regions [138,139,140,141]. Moreover, an increase in GluN2B at extrasynaptic sites on medium-sized spiny striatal neurons has been observed to contribute to phenotype onset [142,143] in a model of HD where synaptic/extrasynaptic NMDAR balance influences the inclusion and toxicity of mutant huntingtin [144]. This may suggest that both GluN2 subunit identity and location influence the progression of HD pathology, however. further investigation is required to establish the contribution of CTD^2B^ mediated signalling.

Impaired NMDAR-mediated neurotransmission has been proposed as one of the contributing factors in schizophrenia (SZ) pathology, owing to the observation that psychomimetic compounds can transiently replicate SZ symptomology by blocking NMDAR neurotransmission [145,146]. Genetic evidence also points to a role for disrupted NMDAR signalling in SZ. For instance, GRIN2A has been implicated as a risk gene for SZ by a recent genome wide association study [147]. In addition, exome sequencing has revealed several de novo SZ associated mutation in both the *GRIN2A* and *GRIN2B* genes, several of which occur in the CTD [42,49]. However, it remains to be seen whether these de novo CTD mutations are causative in SZ. If they do indeed contribute to the pathogenesis of SZ, the question then becomes by what means? Do these mutants disrupt key CTD dependent signalling pathways or do they simply impair trafficking and therefore synaptic NMDAR content. Robust characterisation of these variants will be required to address these key questions.

## 7. Concluding Remarks

Studies are starting to shed light on the role of NMDAR CTDs in both neurodevelopment, acute excitotoxicity, and neurodegeneration. However, despite this, many questions remain unanswered. For instance, while evidence suggests that CTDs are not required for developmental NMDAR compositional changes, the role of CTD sequences in activity-dependent compositional changes remains to be ascertained. Future work using both currently available KI mouse models and KO models of putatively important phosphorylation sites is needed to shed light on the vital mechanisms underpinning this process. Additionally, if the CTD is identified as important in this mechanism then the next task would be to identify how CTD sequences control subunit removal and insertion at the synapse, and whether this process is dependent on phosphorylation events, CTD specific signalling cascades, or whether stability in response to changing activity is different between subunits owing to the recruitment of different protein complexes by distinct CTDs. A better understanding of the mechanisms involved in activity-dependent changes will set the groundwork for identifying how these processes may be aberrant in neurodevelopmental disorders. In the meantime, it would also be beneficial to functionally characterise some of the many CTD variants that have already been identified in ASD, ID and epilepsy patients. 

While this review mainly focuses on GluN2A and GluN2B CTDs, it is important to note that there is also evidence to suggest a role for the influence of GluN2C and GluN2D in neuronal survival and cell death (reviewed by [148]). Both in vitro and In vivo evidence suggests a critical role for GluN2D in NMDA-induced excitotoxicity [149,150,151]. This suggests that GluN2D, much like GluN2B, may preferentially couple to pro-death signalling pathways to mediate cell death in brain regions where GluN2D is expressed. Therefore, it would be of great interest to establish the involvement, if any, of the GluN2D CTD in GluN2D mediated excitotoxicity. Finally, as a goal of understanding the role of GluN2 CTDs in pathophysiology is to identify potential therapeutic targets, it is important to understand how post translational modification of the CTD influences the efficacy of therapeutic drugs. It has already been demonstrated that phosphorylation and palmitoylation state of the CTD can influence the sensitivity of NMDARs to pharmacological intervention [68,152]. Therefore, establishing how particular disease states may influence post translational modification of the CTD will be important in developing optimal therapeutic strategies.

## Figures and Tables

**Table 1:** Summary of findings from DAPK1 studies.

Model	Treatment	Findings	Study
HEK293	Coexpression of GluN1/GluN2B and constitutively active DAPK1	↑ GluN1/GluN2B peak amplitude of receptor currents ↑ S1303 phosphorylation	[103]
DAPK1^+/+^ cortical neurons	OGD	↑ S1303 phosphorylation ↑ amplitude of extrasynaptic NMDA mediated currents and Ca^2+^ transients	[103]
DAPK1^+/+^ cortical neurons	Bath application of NMDA (20 µM and 50 µM) and OGD	No change in s1303 phosphorylation	[104]
DAPK1^−/−^ cortical neurons	OGD	No change in s1301 phosphorylation	[103]
DAPK1^−/−^ in vivo	MCAO	↓ infarct volume as measured by TTC staining	[103]
DAPK1^−/−^ in vivo	MCAO	No change to infarct volume as measured by H-E staining	[104]
DAPK1^+/+^ in vivo	CA/CPR	No change in s1303 phosphorylation	[105]

Note: an up arrow indicates an increase; a down arrow indicates a reduction or decrease.

## Data Availability

Not applicable.
